# Students’ and parents’ attitudes toward basic life support training in primary schools

**DOI:** 10.3325/cmj.2013.54.376

**Published:** 2013-08

**Authors:** Jasna Petrić, Mario Malički, Domagoj Marković, Julije Meštrović

**Affiliations:** 1 Department of Pediatrics, University Hospital Split, Split, Croatia; 2 Department of Research in Biomedicine and Health, School of Medicine, University of Split, Split, Croatia

## Abstract

**Aim:**

To assess attitudes of students and their parents toward basic life support (BLS) training in primary schools, along with their perceptions of students’ fears toward applying and training BLS.

**Methods:**

In October 2011, a specifically designed, voluntary and anonymous questionnaire was distributed to 7th and 8th grade students and to their parents in two primary schools in Split, Croatia. Completed questionnaires were analyzed to determine the validity of the scale, and to determine sex and group differences in individual items and the whole scale.

**Results:**

The questionnaires were completed by 301 school children and 361 parents. Cronbach’s alpha of the whole scale was 0.83, indicating good internal consistency. The students’ score for the whole attitude scale was 73.7 ± 11.1 out of maximum 95, while the parents’ score was 68.0 ± 11.9. Students’ attitude was significantly more positive than that of the parents (U = 29.7, *P* < 0.001). The greatest perceived students’ fear toward applying BLS was that they would harm the person in need of BLS.

**Conclusion:**

Our study showed that in Croatia both students in their last two years of primary school and their parents had a positive attitude toward BLS training in primary schools. Implementing compulsory BLS training in Croatia’s primary schools could help increase students’ confidence, quell their fears toward applying BLS, and possibly even increase the survival of bystander-witnessed cardiac arrests.

Out-of-hospital cardiac arrests (OHCA) globally affect 55 people per 100 000 a year, with an average survival rate of 7% (1). The main factors associated with increased survival rates of those affected by OHCA are timely bystander cardiopulmonary resuscitation (CPR), initial heart rhythm, return of spontaneous circulation, and pre-hospital defibrillation (2). Early bystander CPR slows the progress of ventricular fibrillation’s progression to asystole (3) and is administered on average 4 minutes earlier than CPR performed by emergency medical services (4). However, bystanders attempt CPR in only 32% of cases (4) mostly because lack of training and fear of causing damage, contracting an infectious disease, and not being able to provide adequate resuscitation (5). In 2003, the International Liaison Committee on Resuscitation recommended CPR training in schools (6) and in 2010 the American Heart Association suggested CPR to be a requirement for high school graduation (7). Introduction of CPR and basic life support (BLS) training in schools could lead to acquisition of these skills by the majority of the world’s population and possibly increase the likelihood of survival from OHCA and other emergency conditions.

In Croatia, as in many countries in the world, primary school is the only form of education compulsory for all citizens. It lasts 8 years, with the earliest enrollment age of 6. Research has already shown that children aged 13-14 years can perform chest compressions as well as adults, and that the use of automated external defibrillators can be mastered even at a much earlier age (8,9). This makes children in their final grades (7th and 8th) of primary school ideal candidates for BLS training. In this study, we wanted to assess the attitudes of 7th and 8th graders and their parents toward BLS training in primary schools, as well as both groups’ perceptions of students’ fears toward applying and training BLS.

## Methods

In October 2011, we distributed a specifically designed, voluntary, and anonymous questionnaire (supplementary material(web extra material 1)) to all 7th and 8th grade students of the two largest public primary schools in Split, Croatia. Students were also asked to take two questionnaires home to their parents and to return them within 7 days. Approval for the study was given by our School of Medicine’s Ethics Board and the headmasters of the schools in question.

The questionnaire consisted of demographic data (sex and date of birth) and 27 statements with Likert-type responses ranging from 1 (strongly disagree) to 5 (strongly agree). The statements were constructed by the authors after a literature search on the topic, followed by a brainstorming session. This led to the construction of 40 initial statements, 30 of which were designed to measure the attitude toward BLS training in primary schools and 10 to measure perceived fears. These statements were reviewed by three teachers from our University who advised merging, exclusion, and reformulations of several statements. This left us with 22 items for the attitude scale (11 positive and 11 negative) and 5 items for perceived fears. After the administration of the questionnaire (22 + 5 items), a content examination and discrimination validation was performed, examining inter-item correlations and item-total correlation for 22 items of the attitude scale. This process identified 19 items with item-total correlations of (0.285-0.586) and 3 items with low item-total correlations (0.07-0.09), which implied that the items did not belong to the scale. These items conceptually dealt with the methods of implementation of BLS and not the attitudes toward it, and were therefore excluded from further analysis. The final scale contained 19 items. In calculating the total scale score, we reversed the results of the negative statements. Depending on the distribution of data we used appropriate descriptors. Differences between the two groups, both for the entire scale and individual items were analyzed by Mann-Whitney U Test. The significance level for all statistical tests was 0.05. Data were analyzed using the SPSS, version 19 (SPSS, Inc., Chicago, IL, USA). All questionnaire items were translated into English and back-translated by an independent expert to confirm the validity of the translation.

## Results

A total of 301 (100%) students (143 male and 158 female) and 361 (60%) parents (163 male and 181 female, with data on 17 parents missing) returned the completed questionnaires. Students were 12-15 years old, with a median of 13 (data missing for 12), while their parents were 29-64 years old, with a median of 43 (data missing for 18).

Cronbach’s alpha of the whole scale was 0.83, indicating good internal consistency; with error variance of 0.31. Students’ score for the whole attitude scale was 73.7 ± 11.1 out of maximum 95, while parents’ score was 68.0 ± 11.9; standard error for individual respondents was 4.57 and 4.90, respectively. Both scores differed significantly from the neutral score of 57.0, indicating positive attitudes toward CPR training in primary schools. However, students’ score significantly differed from that of the parents (U = 29.7, *P* < 0.001), indicating that students show a more positive attitude. Both students and parents expressed the strongest agreement with the statement that each and every person should know BLS (67.2% vs 73.9%) and that BLS training would increase the students’ confidence (74.1% vs 67.3%) (Table 1). Significantly more parents believed that BLS training in schools should be performed by medical professionals rather than teachers (U = 42.6, *P* < 0.001), while significantly more students believed that students were both physically (U = 61.3, *P* < 0.001) and mentally (U = 64.1, *P* < 0.001) capable to apply BLS, and that they would use their BLS knowledge to better handle other emergency situations (U = 64.6, *P* < 0.001; Table 1). Sex differences among parents were found only for the item 9 (“By learning BLS students will take more care of their friends”), where the mothers showed a more positive attitude (U = 16.58, *P* = 0.022). Sex differences among students were found for the items 1 (“BLS teaching should commence in primary school*”*) and 7 (“Learning BLS would increase the students’ confidence”), where female students had a more positive attitude (U = 12.45, *P* < 0.001 and U = 12.42, *P* = 0.009, respectively). Parents perceived students’ fears toward applying BLS to be greater than students perceived them to be and the greatest fear in both groups was that of harming the person while performing BLS (Table 2). No sex differences were found regarding fears toward applying BLS (data not shown).

**Table 1:** Attitudes of students (S, n = 301) and their parents (P, n = 361) toward basic life support (BLS) training in primary schools*

Statement	Strongly disagree		Disagree		Neither agree nor disagree		Agree		Strongly agree		*P*
	S	P	S	P	S	P	S	P	S	P	
1. BLS training should commence already in primary schools.	3.7	8.7	4.3	4.7	15.0	16.5	19.7	19.6	57.3	50.6	0.028
2. The best place to teach students BLS is school, and not a medical institution.	11.2	20.8	10.2	9.4	26.4	25.0	18.0	15.0	34.2	29.7	0.012
3. BLS training for students should be done by school teachers, and not by medical professionals.	31.8	51.3	20.6	13.4	25.3	20.6	8.8	7.5	13.5	7.2	<0.001
4. School teachers need to know BLS.	6.4	9.8	5.0	7.8	12.0	14.0	13.0	11.8	63.5	56.6	0.027
5. School teachers should be competent to teach BLS.	11.1	9.5	8.1	6.4	35.6	19.0	20.5	15.7	24.8	49.3	<0.001
6. School teachers are not willing to teach BLS.	11.1	12.6	8.1	8.6	35.6	40.6	20.5	11.1	24.8	27.1	0.354
7. Learning BLS would increase students’ confidence.	3.7	4.5	2.7	3.9	7.4	9.8	12.1	14.5	74.1	67.3	0.058
8. By learning BLS students will be able to avoid risk behaviors.	6.4	10.0	6.1	7.2	15.5	20.9	21.9	24.5	50.2	37.3	0.001
9. By learning BLS students will take more care of their friends.	5.7	6.7	5.7	6.1	19.1	24.6	23.7	22.6	45.8	39.9	0.078
10. By learning BLS students will be able to better handle emergency situations.	15.2	18.3	7.4	11.4	6.4	18.1	13.8	16.7	57.2	35.6	<0.001
11. Students are not overburdened to prevent them from learning BLS.	8.4	14.2	7.4	9.2	23.3	28.4	26.0	14.5	34.8	33.7	0.021
12. In the students’ timetable there is room for Basic Life Support.	17.1	21.9	9.7	10.2	18.8	25.2	18.1	13.6	36.2	29.1	0.012
13. Students are mentally capable to apply BLS methods to people in need.	7.0	10.8	10.4	14.2	15.8	27.8	17.8	13.6	49.0	33.6	<0.001
14. Students are physically capable to apply chest compressions to people in need.	9.9	10.0	7.8	12.2	16.7	29.4	16.7	12.7	49.0	35.7	<0.001
15. Learning BLS in schools is supported by parents.	7.3	11.9	3.8	10.4	15.6	33.6	23.5	9.9	49.8	34.2	0.034
16. Learning BLS in schools is supported by the public.	9.3	16.1	5.5	9.7	26.0	34.6	18.0	12.3	41.2	27.3	<0.001
17. Everyone should know how to apply BLS.	5.1	2.9	2.0	3.7	11.3	6.9	14.3	12.6	67.2	73.9	0.057
18. It is necessary that parents teach their children BLS.	21.0	22.7	11.7	14.7	22.7	25.3	15.8	15.5	28.9	21.8	0.069
19. More people would be able to apply BLS if everyone learned it at school.	3.1	4.6	5.5	3.5	12.7	15.6	17.5	22.2	61.0	54.2	<0.001

*Numbers given are percentages.

**Table 2:** Perceptions of students (S) and their parents (P) toward fears regarding basic life support (BLS) training in primary schools*

Statement	Strongly disagree		Disagree		Neither agree nor disagree		Agree		Strongly agree		*P*
	S	P	S	P	S	P	S	P	S	P	
1. Students are not afraid of contagious diseases they can get from manikins used for BLS.	20.2	22.7	11.1	14.7	23.9	25.3	16.2	15.5	28.6	21.8	0.003
2. Students are not afraid to apply BLS despite infections they may get from the person in need.	8.4	13.9	17.6	16.4	31.1	36.8	19.3	15.0	23.6	17.3	0.013
3. Students are not afraid to apply BLS despite the potential harm they may cause to the one in need.	10.9	13.2	17.7	9.2	39.9	40.8	15.7	16.4	15.7	20.4	0.091
4. Students are not afraid to apply BLS despite their lack of knowledge of the technique.	10.0	6.7	8.2	5.2	19.2	15.9	24.7	23.2	37.8	49.0	0.002
5. More people would be willing to apply BLS if its training only involved chest compressions without mouth to mouth or mouth to nose resuscitation.	9.6	16.7	8.9	7.5	27.1	31.1	25.7	17.6	28.8	27.1	0.034

*Numbers given are percentages.

## Discussion

Our study showed that both students and their parents had a positive attitude toward BLS training in primary schools and that they most strongly believed it would greatly benefit students’ self-confidence. A more positive attitude of female students toward BLS training and its impact on students’ self-confidence warrants further investigation, but possibly originates from the previously reported lower levels of self-confidence in female students (10). Sex difference between parents regarding possible impact that BLS training can have on students’ relationship with their friends also warrants further investigation, but it most likely originates from the observed sex differences in parents’ involvement in facilitation of their child’s social relationships (11). The greatest fear expressed by the students was that of harming the person in need of BLS, which if not quelled in time can possibly lead toward restraint or failure to provide help. Both students and parents reported that they would prefer that the BLS is taught by medical personnel rather than school teachers. We believe that such opinion is related to the public perception of BLS as a set of complicated skills that should be taught by personnel with real-life experience. Such fears could be countered through wide-spread training and pubic campaigns.

With the rate of more than 8% of cardiac arrests witnessed by students (12), proven psychological and physical readiness of 13-14 year-olds, positive correlations between BLS training and willingness to apply it, and proven cost-efficient training methods, we believe that compulsory BLS training should be implemented in primary schools in Croatia, and in all countries with equivalent length of compulsory education. There were no significant differences in BLS knowledge and skill retention between biannual vs annual repeated training (13), however there is still a lack of longer longitudinal studies on this issue (14). It is possible that annual technique repetition through online media and self-learning kits, public television campaigns, and primary health care pamphlets and advertisements, with obligatory BLS testing at graduation from primary and secondary schools or colleges and universities would be enough to complement initial BLS training. Courses and testing could also be implemented alongside workplace safety classes for adults.

A limitation of our study is that sampling was not randomized, and it only included students in public schools of one city in Croatia. Therefore, it is possible that a larger or more diverse sample could have yielded different results. The attitudes of students and their parents could also be subject to family experiences with cardiac arrests or other illnesses, previously experienced accidents, as well as social and cultural differences. We also did not measure the willingness of students and their parents to perform BLS in real-life situations; however studies have shown that BLS training greatly improves both the willingness and the rates of bystander-administered BLS (15-17). Implementing compulsory BLS training in primary schools could help subside students’ fears, increase their confidence, and globally help increase survival from OHCA.
