# Supplementary material for: Students’ and parents’ attitudes toward basic life support training in primary schools
Source: Croat Med J. 2013 Aug;54(4):376–80. doi: 10.3325/cmj.2013.54.376 (PMC3760662; doi:10.3325/cmj.2013.54.376)
Supplement: Supplementary material [file CroatMedJ_54_s005.pdf]

Supplementary data - Questionnaire

QUESTIONNAIRE ON TEACHING BASIC LIFE SUPPORT IN PRIMARY SCHOOLS

This anonymous questionnaire was designed in order to research the need for teaching Basic Life Support (BLS) in primary schools in Split.
Please answer each question carefully and honestly.

Year of Birth: __________ **Sex:** M F

Using the following scale please circle the number that corresponds to your agreement with the following statement:

Strongly disagree	Disagree	Neither agree nor disagree	Agree	Strongly agree
1	*2*	*3*	*4*	*5*

**1.** Basic Life Support training should already commence in primary school.	1	2	3	4	5
**2.** The best place to teach 7th and 8th graders Basic Life Support is a school, and not a medical institution.	1	2	3	4	5
**3.** Basic Life Support should be taught to 7th and 8th graders by school teachers, and not by doctors or other medical personnel.	1	2	3	4	5
**4.** School teachers need not to know Basic Life Support.	1	2	3	4	5
**5.** School Teachers should be able to teach Basic Life Support.	1	2	3	4	5
**6.** School Teachers are not willing to teach Basic Life Support.	1	2	3	4	5
**7.** Learning Basic Life Support would reduce students' self-confidence.	1	2	3	4	5
**8.** By learning Basic Life Support students will be able to avoid risk behaviors.	1	2	3	4	5
**9.** By learning Basic Life Support students will take more care of their friends.	1	2	3	4	5
**10.** By learning Basic Life Support students will not be able to better handle emergency situations.	1	2	3	4	5
**11.** Students are overburdened and they should not have to learn Basic Life Support as well.	1	2	3	4	5
**12.** In the students' timetable there is no room for Basic Life Support.	1	2	3	4	5
**13.** 7th and 8th graders are not MENTALLY capable of providing help (applying BLS methods) to people in need.	1	2	3	4	5
**14.** 7th and 8th graders are not PHYSICALY able to provide chest compressions to people in need.	1	2	3	4	5

**15.** Learning Basic Life Support in schools is not supported by parents.	1	2	3	4	5
**16.** Learning Basic Life Support in schools is not supported by the public.	1	2	3	4	5
**17.** Everyone should know how to apply Basic Life Support.	1	2	3	4	5
**18**. It is not necessary that parents teach their children Basic Life Support.	1	2	3	4	5
**19.** More people would be able to apply Basic Life Support if everyone learned it at school.	1	2	3	4	5
**20.** More people would be willing to apply Basic Life Support if its training only involved chest compressions without mouth to mouth or mouth to nose resuscitation.	1	2	3	4	5
**21.** Students are not afraid of contagious diseases they can get from the training manikins.	1	2	3	4	5
**22.** Students are not afraid to apply Basic Life Support despite possible infections they can get from the person in need.	1	2	3	4	5
**23.** Students are not afraid to apply Basic Life Support despite the potential harm they may cause to the person in need.	1	2	3	4	5
**24.** Students are afraid to apply Basic Life Support because they lack knowledge of the same.	1	2	3	4	5
